# Assessment of functioning in patients with schizophrenia and schizoaffective disorder with the Mini-ICF-APP: a validation study in Italy

**DOI:** 10.1186/s13033-015-0030-x

**Published:** 2015-10-31

**Authors:** Federica Pinna, Andrea Fiorillo, Massimo Tusconi, Beatrice Guiso, Bernardo Carpiniello

**Affiliations:** Department of Public Health, Clinical and Molecular Medicine-Unit of Psychiatry, University of Cagliari, Via Liguria 13, 0917 Cagliari, Italy; Department of Psychiatry, University of Naples SUN, Naples, Italy

**Keywords:** Mini-ICF-APP, Validity, Outpatients, Schizophrenia, Schizoaffective disorders

## Abstract

**Background:**

The aim of the study was to evaluate validity of the Italian Mini-ICF-APP (Mini-ICF Rating for Limitations of Activities and Participation in Psychological Disorders) in schizophrenia and related disorders.

**Methods:**

74 outpatients affected by schizophrenia or schizoaffective disorders attending a University-based community mental health centre were recruited to the study. All participants underwent comprehensive evaluation using standardized instruments to assess clinical, neurocognitive and functional status. Concurrent validity of Mini-ICF-APP was evaluated and compared to severity scores obtained using the Clinical Global Impression-Schizophrenia scale (CGI-SCH), Positive and Negative Syndrome scale (PANSS), Mini Mental State Examination test (MMSE), Brief Assessment of Cognition in Schizophrenia scale (BACS) and Personal and Social Performance scale (PSP). Construct validity was evaluated by comparing scores obtained at Mini-ICF-APP by remitted versus non-remitted patients, and by recovered versus unrecovered patients. Discriminant validity was evaluated comparing scores on Mini-ICF-APP and Subjective Well-being (SWN) scale. Results: the total score and 12 out of the 13 Mini-ICF-APP items correlated significantly with total score at PSP; Mini-ICF-App total score was moreover significantly correlated with total scores at CGI-SCH, PANSS, MMSE, as well as with several BACS items. Total scores obtained at Mini-ICF-APP were significantly higher among remitted and recovered patients. No relevant correlations were found between scores of Mini-ICF-APP and SWN scales.

**Results:**

The total score and 12 out of the 13 Mini-ICF-APP items correlated significantly with total score at PSP; Mini-ICF-App total score was moreover significantly correlated with total scores at CGI-SCH, PANSS, MMSE, as well as with several BACS items. Total scores obtained at Mini-ICF-APP were significantly higher among remitted and recovered patients. No relevant correlations were found between scores of Mini-ICF-APP and SWN scales.

**Conclusion:**

the Italian version of Mini-ICF-APP is a valid instrument for use in evaluating functioning in chronic patients with schizophrenia and related disorders.

## Background

Among all mental disorders, schizophrenia is undoubtedly the most frequently related to the development of disability [1]. Indeed, approx. 80 % of adults affected by schizophrenia and related disorders have been estimated to show persistent deficits in personal and social functioning [2]. Although clinical remission [3] is seen as the main goal of treatment, nowadays recovery, which implies a satisfactory level of functioning [4], is viewed as the ultimate target. Thus, the importance of personal and social functioning is of course indisputable, also bearing in mind that functioning deficits are comprised among the basic diagnostic criteria for schizophrenia and other mental disorders. However, in clinical practice the routine assessment of functioning is still uncommon for a series of reasons, likely including the lack of a simple, user-friendly rating scale for use as a reference tool; however, on the international scenario [5] a series of reliable and valid instruments such as GAF [6], SOFAS [7], PSP [8], and WHO-DAS2 [9] are available. In this context, the Mini-ICF-APP, specifically designed in line with parameters of the World Health Organization International Classification of Functioning, Disability and Health (ICF) for use in mental health settings, was first developed and validated in Germany [10, 11] and recently translated and validated in English [12] and Italian [13]. The original German version was validated on a sample of patients affected by non-psychotic mental disorders, whilst both the English and Italian versions were validated using mixed patient samples, including a limited number of subjects with schizophrenia or other psychotic disorders. Accordingly, the present study was designed with the aim of validating the instrument for use in schizophrenia and schizoaffective disorders, in the context of an ongoing observational, prospective study on remission and recovery in chronic psychotic patients [14].

## Methods

### Sample

This cross-sectional study was conducted on a convenience sample of the first 74 outpatients affected by schizophrenia or schizoaffective disorders according to DSM-IV-TR, who attended a University-based community mental health centre (CMHC) and had previously concluded a two-year follow up period in the above cited observational study [14]. Patients with comorbid psychiatric and/or physical disorders were included in the study, with the exception of those affected by mental retardation or severe organic brain diseases. Subjects underwent standard care routinely available in Italian CMHCs (clinical monitoring at least on a monthly basis; pharmacological treatment; home care when required, psychosocial and rehabilitation interventions tailored to patient’s needs). The study was approved by the Ethics Committee of the Local Health Authorities in Cagliari (Italy) and was conducted in compliance with national laws. All patients gave their informed consent to take part in the study. The sample included patients affected by schizophrenia (N = 32, 43 %) or schizoaffective disorders (N = 42, 57 %). The sociodemographic characteristics of the sample are reported in Table 1; clinical characteristics are reported in Table 2. The sample included patients who were mostly male (70 %), with a mean age of 43.5 years, single (85 %), unemployed (77 %), and with a good level of education (mean years of education = 11). Patients were characterized by early age at onset of the illness (approx. 23 years), a long clinical history (mean duration approx. 15 years), and a continuous course of the disorder (approx. 57 % of cases); all patients had been prescribed psychopharmacological treatment.

**Table 1:** Sociodemographic characteristics of the sample (N = 74)

Characteristics	Statistics
Gender N (%)	
Males	52 (70.3)
Females	22 (29.7)
Age	
Mean ± SD	43.46 (10.06)
Range	25–68
Education	
Mean years ± SD	10.67 (3.88)
Range	4–14
Marital status N (%)	
Singles	63 (85.1)
Married	11 (14.9)
Occupation N (%)	
Employed	17 (23)
Unemployed	57 (77)

**Table 2:** Clinical characteristics of the sample

Characteristics	Statistics
Diagnosis N (%)	
Schizophrenia	32 (43.2)
Schizoaffective disorders	42 (56.8)
Age at onset	
Mean ± SD	22.83 (10.06)
Range	13–53
Duration of untreated psychosis	
Mean months ± SD	26.05 (53.85)
Range	24–564
Comorbidities N (%)	
Anxiety disorders	7 (9.5)
Personality disorders	8 (11.0)
Alcohol abuse/dep	9 (12.3)
Other subst abuse/dep	16 (21.9)
Previous clinical course N (%)	
Episodic with remission	11 (14.9)
Episodic without remission	17 (23.0)
Continuous	42 (56.8)
Non specified	4 (5.4)
Previous hospital adm N (%)	
Yes	47 (63.5)
No	27 (36.5)
Previous suicide attempts N (%)	
Yes	20 (27)
No	54 (73)
Previous legal problems N (%)	
Yes	6 (8.1)
No	68 (91.9)
Treatments N (%)	
Antipsychotics	74 (100.0)
Mood stabilizers	17 (23.0)
Antidepressants	19 (25.7)
Anxiolytics	42 (56.7)
Other	13 (17.6)
Psychotherapy	9 (12.1)
Rehabilitation programmes	16 (21.6)
Remission N (%)	
Clinical	40 (54.1)
Functional	22 (29.7)
Clinical + functional (recovery)	20 (27.0)

### Ratings

Evaluation was performed by residents in psychiatry using a set of standardized methods, after adequate training in the use of all adopted instruments. Personal and social data, and clinical history were collected in a structured interview purpose-developed for the study. After providing informed consent, patients were interviewed using the Italian versions [15, 16] of SCID-I [17] and SCID-II [18]; inter-rater reliability, assessed using Cohen’s K before the study, was higher than 0.80. Symptom severity was evaluated using the Italian version [19] of PANSS [20]^,^ consisting of 30 items grouped into three distinct symptom clusters (positive, negative, general psychopathology) rated on a 7 point scale; an accurate picture of symptomatology is generally based on summary score obtained for each symptom cluster and on total score. Interviews were conducted by means of the Italian version [21] of the Structured Clinical Interview for Positive and Negative Scale (SCI-PANSS) [22]. Ratings were based on criteria indicated in the PANSS Manual [23] and, wherever possible, PANSS assessment included a standard section of queries addressed to treating clinicians and caregivers. The global clinical status was also evaluated by CGI-SCH [24], the version of the CGI (clinical global impression scale) adapted for use in Schizophrenia; the CGI-SCH provides for the global assessment of positive, negative, cognitive, and depressive symptoms and overall clinical condition over the week before the visit; CGI takes three aspects into account: severity of symptoms, global improvement and an efficacy index. For the purposes of this study, only the severity scale (CGI-SCHs) was used; scores ranged from 1 (no symptoms) to 7 (most severe symptoms). Cognitive functioning was evaluated by means of BACS [25], a scale comprising a series of cognitive dimensions, namely list learning, digit sequencing, category instances and controlled oral word association test, symbol coding and executive functions; a gender/age/education adjusted score, and subsequently an equivalent score were calculated [26]. MMSE [27] was also administered, calculating an age/education adjusted score [28]. Functioning was evaluated by PSP [8], assumed as a gold standard, to assess social functioning of patients in four main areas: socially useful activities, personal and social relationships, self-care and disturbing/aggressive behaviours. Each area was evaluated on a six-point scale, where lower ratings indicate better functioning. A comprehensive overall score ranging from 1 (maximum dysfunction) to 100 (maximum functioning) was attributed, based on scores obtained at each single area. A total score exceeding 70 indicates a condition of “functional remission”, with scores related to overall good functioning. A non-standardized interview was conducted with the patient, caregivers (when available) and with the treating physician, with the aim to assess functioning by means of PSP. Patients were also submitted to the Subjective Well-being under Neuroleptic scale (SWN-S) [29], a self-administered rating scale devoted to evaluating the psychological and physical well-being of patients treated with neuroleptics. For the purpose of the study the short version (20 items, including 5 subscales: mental functions, self-control, physical function, emotional control, social capability) was used. The Mini-ICF-APP [10, 11] is a clinician-rated instrument developed to measure limitations of capacity in people with mental disorders; these limitations are rated in terms of competence throughout thirteen domains, namely adherence to regulations, planning and structuring of tasks, flexibility, competency, endurance, assertiveness, contact with others, group integration, intimate relationships, non-work activities, self-care, mobility and competence to judge and decide. Each dimension is rated on a five-point Likert scale (0 = no impairment; 5 = total disability). Ratings are based on information provided by patients, carers, clinical records, as well as direct observation of subjects and on anchor definitions of each item described in the Italian version of the Manual [30].

Inter-rater reliability of PANSS evaluations in terms of ICC (Intraclass correlation coefficient) ranged from 0.65–0.95 for the PANSS total score, from 0.72–0.97 or the CGI-SGH total score, from 0.79–0.94 for the PSP total score and from 0.81–0.93 for Mini-ICF-APP total score.

### Validity

Concurrent validity was evaluated calculating the correlations between scores at Mini-ICF-APP and PSP, the latter being considered the standard of reference for evaluation of personal and social functioning; moreover, correlations of scores obtained at Mini-ICF-APP and PANSS, CGI-SCHs, BACS, MMSE were calculated, based on the hypothesis of an existing, significant correlation between results relating to symptoms and cognitive status and functioning (i.e. the lower the scores obtained at clinical and cognitive scales, the lower the score at Mini-ICF-APP). Construct validity was evaluated by means of known-groups technique, which involves administering the measurement instrument to groups expected to differ due to known characteristics. Thus Mini-ICF-APP was evaluated by comparing differences in mean scores at each single item and total score obtained by “remitted” and “non remitted” patients and “recovered” and “unrecovered” subjects at the time of evaluation (24 months). The working hypothesis was based on the assumption that both remission and recovery should be associated with achievement of better results at Mini-ICF-APP, i.e. lower mean scores. Clinical remission was assessed using the schizophrenia remission working group criteria (SRWG-cr) [30], based on ratings at eight focal symptoms in positive, negative and general psychopathology subscales of PANSS (P1, P2, P3, N1, N4, N6, G5, G9); patients were judged to be in clinical remission according to a severity criterion (scores obtained at each of these items were required to be ≤3 points, indicating mild severity of symptoms). Due to the cross-sectional nature of the study, clinical remission was evaluated on the basis of severity criterion alone, excluding the duration criterion (remission maintained for 6-months). With regard to functional remission, patients with a PSP total score >70, i.e. cut-off score indicating subjects devoid of significantly impaired functioning [8], were deemed to be remitted. Patients’ overall clinical status was taken into account, and assessments were carried out to determine whether or not they could be considered as “recovered”; for this purpose, due to the lack of a univocal definition for recovery [31, 32], patients characterized by both clinical and functional remission were taken as being recovered. Discriminant validity was evaluated calculating the correlations between scores at Mini-ICF-APP and SWN-S, a scale specifically devoted to evaluate subjective-well being of patients undergoing antipsychotic treatment, on the basis of the hypothesis that no significant correlation should be found between results relating to functioning and treatment-related well-being measures.

### Statistical analysis

Categorical data were analyzed using Pearson’s χ2 test or Fisher’s exact test; continuous variables were assessed by means of Student’s “t” test for independent samples. The magnitude of differences in mean scores obtained at different rating scales used in the study was calculated by means of Cohen’s “d”. Correlations between continuous variables were calculated the Pearson’ r coefficient. Data analyses were performed using SPSS 19.0. Level of significance was set at a p value ≤0.05 for two-tailed hypothesis.

## Results

Ratings at PANSS, CGI-SCHs, BACS, MMSE and PSP are reported in Table 3. Mean scores obtained at clinical and cognitive scales show low-moderate levels of severity and impairment, as expected in a cohort of relatively stable patients in a community treatment setting. No significant gender or diagnosis differences were detected for sociodemographic and clinical variables. The median score at Mini-ICF-APP is generally comprised between 1 and 2 for the majority of items, thus indicating a low-moderate level of impairment (Table 4); no differences were found in mean scores according to gender and diagnosis. With regard to correlational analyses, Mini-ICG-APP total score displayed a significant correlation with PSP total score and scores at the PSP subscales “socially useful activities”, “personal and social relationship” and “self-care”; all Mini-ICF-APP items correlated significantly with PSP total score, with the exception of the “Mobility” item; 4 out of 13 items of Mini-ICF-APP (Adherence to regulations, Structuring of tasks, Flexibility, Self-Care) featured a significant correlation with scores obtained at the PSP subscales “socially useful activities”, “personal and social relationship” and “self-care”, while 8 out of 13 items of Mini-ICF-APP (Competency, Endurance, Assertiveness, Contact with Others, Group Integration, Intimate Relations, Spontaneous activity, Judgement) correlated significantly with 2 out of 4 PSP subscales, namely “Socially Useful Activities”, “Personal and Social Relationship”; only one Mini-ICF-APP item (mobility) was devoid of any correlation with PSP subscales (Table 5). In the symptomatology domain, Mini-ICF-APP total score significantly correlated with scores at CGI-SCHs cognitive and depressive subscales and with the overall scale; moreover, a correlation was observed with PANSS total score and negative and general psychopathology scales (Table 6). With regard to cognition, Mini-ICF-APP total score significantly correlated with MMSE score, and with scores at BACS scales “Verbal memory”, “Verbal fluency” and “Tower of London” (Table 7).

**Table 3:** Rating scales mean scores

Rating scale	Mean value (SD)	Median	Range
CGI SCHs positive symptoms	2.27 (1.43)	2.00	1–6
CGI SCHs negative symptoms	2.78 (1.40)	3.00	1–6
CGI SCHs depressive symptoms	2.20 (1.08)	2.00	1–6
CGI SCHs cognitive symptoms	2.69 (1.43)	3.00	1–7
CGI SCHs total score	3.12 (1.22)	3.00	1–6
PANSS positive	10.36 (3.60)	9.00	7–25
PANSS negative	14.51 (6.93)	12.50	7–36
PANSS general psychopathology	26.51 (8.84)	23.00	16–47
PANSS total score	51.39 (17.0)	47.00	30–91
MMSE	26.04 (3.90)	27.00	11–30
BACS list learning	14.57 (11.80)	11.57	2.75–53.25
BACS digit sequencing task	11.26 (5.88)	11.17	0.25–28.25
BACS verbal fluency/category istances	10.87 (5.24)	10.00	1.00–23.25
BACS semantic fluency/controlled oral word test	15.38 (5.65)	14.50	1.00–34.25
BACS symbol coding	26.13 (13.57)	23.25	0.75–65.75
BACS tower of London	9.36 (7.15)	6.25	0–22.50
PSP socially useful activities	2.54 (1.32)	3.00	0–5
PSP person soc relat	2.39 (1.22)	2.00	0–5
PSP selfcare	0.81 (1.1)	0.00	0–4
PSP dist aggress behav	0.39 (0.79)	0.00	0–3
PSP total	55.65 (16.24)	60.00	18–90

*CGI-SCHs* clinical global impression schizophrenia severity; *PANSS* positive and negative symptoms scale; *BACS* brief assessment cognition scale; *MMSE* mini mental state examination; *PSP* personal and social performance scale

**Table 4:** Mini-ICF-APP mean/median scores

Item	Mean value (SD)	Median	Range
Adherence to regulations	0.89 (1.07)	1.00	0–4
Structuring of tasks	1.57 (1.23)	1.00	0–4
Flexibility	1.99 (1.14)	2.00	0–4
Competency	2.01 (1.20)	2.00	0–4
Endurance	1.89 (1.34)	2.00	0–4
Assertiveness	1.50 (1.21)	1.00	0–4
Contact with others	1.62 (1.22)	1.50	0–4
Group integration	1.62 (1.27)	2.00	0–4
Intimate relations	1.89 (1.25)	2.00	0–4
Spontaneous activity	1.78 (1.17)	2.00	0–4
Self-care	0.70 (0.84)	0.50	0–3
Mobility	0.85 (1.15)	0.00	0–4
Judgement	1.74 (1.12)	2.00	0–4
Total score	20.07 (11.77)	18.50	0–46

**Table 5:** Correlations between Mini-ICF-APP and PSP scores

	PSP socially useful activities	PSP personal and social relationships	PSP selfcare	PSP aggressive and disturbing behaviours	PSP total score
Mini ICF adherence to regulations	0.342**	0.305**	0.303**	0.181	−0.426**
Mini ICF structuring of tasks	0.305**	0.414**	0.268*	0.205	−0.389**
Mini ICF flexibility	0.367**	0.406**	0.242*	0.112	−0.383**
Mini ICF competency	0.460**	0.360**	0.182	0.038	−0.452**
Mini ICF judgement	0.352**	0.393**	0.117	0.146	−0.355**
Mini ICF endurance	0.496**	0.385**	0.109	0.105	−0.459**
Mini ICF assertiveness	0.402**	0.403**	0.157	0.036	−0.376**
Mini ICF contact with others	0.339**	0.447**	0.162	0.141	−0.394**
Mini ICF group integration	0.433**	0.423**	0.147	0.123	−0.417**
Mini ICF intimate relations	0.368**	0.459**	0.178	0.169	−0.481**
Mini ICF spontaneous activity	0.331**	0.384**	0.183	0.196	−0.474**
Mini ICF self-care	0.417**	0.408**	0.314**	0.137	−0.465**
Mini ICF mobility	0.152	0.139	0.120	−0.055	−0.163
Mini ICF total score	0.475**	0.491**	0.241*	0.152	−0.520**

* p < 0.05, ** p < 0.01

**Table 6:** Correlations between Mini-ICF-APP total score, CGI-SCH and PANSS ratings

CGI-SCH positive symptoms	CGI-SCH negative symptoms	CGI-SCH depressive symptoms	CGI-SCH cognitive symptoms	CGI-SCH total score	PANSS positive scale	PANSS negative scale	PANSS general psycho pathology scale	PANSS total score
0.062	0.305**	0.325**	0.248*	0.455**	0.189	0.389**	0.276*	0.342**

* p = 0.05, ** p = 0.01

**Table 7:** Correlations between Mini-ICF-APP total scores, MMSE and BACS

MMSE	BACS list learning	BACS digit sequencing task	BACS verbal fluency/category instances	BACS semantic fluency/controlled oral word test	BACS symbol coding	BACS ower of London
−0.260*	−0.237*	0.215	−0.390**	−0.225	−0.146	−0.277*

* p < 0.05, ** p < 0.01

On assessing construct validity, total mean scores of clinically remitted patients on Mini-ICF-APP were found to be significantly higher than those of unremitted patients; moreover, clinically remitted patients obtained significantly higher mean scores at 10 out of 13 Mini-ICF-APP items (Table 8). Similarly, total mean scores obtained by functionally remitted subjects at Mini-ICF-APP were significantly higher than those of unremitted patients, with significantly higher mean scores being attained in 9 out of 13 items compared to functionally unremitted patients (Table 9). Mean total Mini-ICF-APP scores rated by recovered patients were significantly higher than those of unrecovered subjects, with similar results being registered in 4 out of 13 items of the scale (Table 10). Finally, no significant correlations were found between scores at Mini-ICF-APP and SWN-S scales with the only exception of the ICF “persistence” scale, showing a significant negative correlation with SWN-S Self-control (r = −0.361, p < 0.01), Physical Functioning scales (r = −0.301, p < 0.01) and with SWN-total score (r = −0.305, p < 0.01).

**Table 8:** Mini-ICF-APP mean scores ± SD according to clinical remission status

Item	Remitted (N = 40)	Non remitted (N = 34)	Cohen’s d	t value (df)	p value
Adherence to regulations	0.70 (0.99)	1.12 (1.12)	−0.379	−1.682 (66.5)	0.094
Structuring of tasks	1.15 (1.23)	2.06 (1.04)	−0.798	−3.438 (72)	0.001
Flexibility	1.67 (1.22)	2.35 (0.92)	−0.629	−2.651 (72)	0.010
Competency	1.75 (1.22)	2.32 (1.09)	−0.492	−2.188 (72.00)	0.038
Endurance	1.57 (1.30)	2.26 (1.31)	−0.528	−2.266 (69.89)	0.027
Assertiveness	1.32 (1.16)	1.71 (1.24)	−0.324	−1.352 (68.34)	0.181
Contact with others	1.32 (1.18)	1.97 (1.19)	−0.506	−2.327 (69.90)	0.023
Group integration	1.22 (1.25)	2.09 (1.14)	−0.727	−3.107 (71.64)	0.003
Intimate relations	1.50 (1.21)	2.35 (1.12)	−0.729	−3.127 (72)	0.003
Spontaneous activity	1.37 (1.17)	2.26 (0.99)	−0.821	−3.537 (72)	0.001
Self-care	0.55 (0.68)	0.88 (0.98)	−0.391	−1.671 (57.36)	0.10
Mobility	0.82 (1.15)	0.88 (1.17)	−0.050	−0.211 (69.40)	0.83
Judgement	1.50 (1.15)	2.03 (1.03)	−0.485	−2.085 (71.82)	0.041
Total score	16.47 (11.84)	24.29 (10.33)	−0.703	−3.033 (71.94)	0.003

**Table 9:** Mini-ICF-APP mean scores ± SD according to functional remission status

Item	Remitted (N = 22)	Non remitted (N = 52)	Cohen’s d	t value (df)	p value
Adherence to regulations	0.54 (0.80)	1.04 (1.13)	−0.510	−2.122 (55.5)	0.038
Structuring of tasks	1.18 (1.33)	1.73 (1.16)	−0.440	−1.683 (35.06)	0.101
Flexibility	1.54 (1.22)	2.17 (1.06)	−0.551	−2.095 (35.03)	0.043
Competency	1.64 (1.40)	2.17 (1.07)	−0.425	−1.608 (32.08)	0.118
Endurance	1.18 (1.29)	2.19 (1.25)	−0.795	−3.096 (38.42)	0.004
Assertiveness	1.23 (1.23)	1.61 (1.19)	−0.314	−1.251 (38.41)	0.218
Contact with others	1.23 (1.30)	1.79 (1.16)	−0.454	−1.744 (35.71)	0.090
Group integration	1.14 (1.21)	1.83 (1.25)	−0.560	−2.227 (40.85)	0.032
Intimate relations	1.23 (1.23)	2.17 (1.15)	−0.789	−3.078 (37.25)	0.004
Spontaneous activity	1.32 (1.29)	1.98 (1.07)	−0.556	−2.122 (34.02)	0.041
Self-care	0.41 (0.66)	0.83 (0.87)	−0.543	−2.232 (51.79)	0.030
Mobility	0.82 (1.33)	0.86 (1.08)	−0.030	−0.147 (33.36)	0.884
Judgement	1.41 (1.18)	1.88 (1.08)	−0.415	−1.623 (36.54)	0.113
Total score	14.86 (12.02)	22.27 (11.05)	−0.641	−2.479 (36.76)	0.018

**Table 10:** Mini-ICF-APP mean scores ± SD according to recovery status

Item	Recovered (N = 20)	Non recovered (N = 54)	Cohen’s d	t value (df)	p value
Adherence to regulations	0.55 (0.82)	1.02 (1.12)	−0.478	−1.954 (46.24)	0.057
Structuring of tasks	1.15 (1.38)	1.72 (1.14)	−0.450	−1.650 (29.04)	0.110
Flexibility	1.50 (1.23)	2.16 (1.06)	−0.574	−2.139 (29.96)	0.041
Competency	1.65 (1.46)	2.15 (1.07)	−0.390	−1.604 (72)	0.113
Endurance	1.30 (1.30)	2.11 (1.29)	−0.625	−2.382 (33.92)	0.023
Assertiveness	1.30 (1.26)	1.57 (1.19)	−0.220	−0.843 (32.37)	0.406
Contact with others	1.30 (1.34)	1.74 (1.17)	−0.349	−1.298 (30.32)	0.204
Group integration	1.10 (1.25)	1.81 (1.23)	−0.572	−2.191 (33.46)	0.036
Intimate relations	1.30 (1.26)	2.11 (1.18)	−0.663	−2.502 (32.03)	0.018
Spontaneous activity	1.30 (1.34)	1.96 (1.06)	−0.546	−1.990 (28.32)	0.056
Self-care	0.45 (0.68)	0.79 (0.87)	−0.435	−1.781 (43.25)	0.082
Mobility	0.90 (1.37)	0.83 (1.07)	0.056	−0.196 (28.13)	0.846
Judgement	1.40 (1.23)	1.87 (1.06)	−0.409	−1.512 (30.16)	0.141
Total score	15.20 (12.46)	21.87 (11.08)	−0.565	−2.105 (30.82)	0.044

## Discussion

The patient sample studied provided an adequate representation of chronic patients with schizophrenia and related disorders generally attending community mental health centres in Italy. Indeed, the sample was made up prevalently by middle-aged, single, unemployed, fairly well educated males, with a long duration of illness, early age at onset, and continuous course of the disorder; ratings obtained at CGI-SCH, PANSS, MMSE, BACS and MMSE, relating to symptoms, cognition and functioning, denote a fairly stable clinical condition characterized by a low/moderate symptoms’ severity, cognitive impairment and social disability, as expected in a cohort of relatively stable, chronic patients in a community setting. Indeed, approximately 50 % of our patients were in clinical remission, slightly less than one-third in functional remission, and about one quarter viewed as recovered in line with the adopted criteria, a finding largely consistent with previous data published by our group [14, 33] and with literature findings [34–37]. Mean and median scores obtained at Mini-ICF-APP were generally comprised between 1 and 2 for the majority of items, thus indicating a low-moderate level of impairment, with only two items (self-care and mobility) showing very low scores (both means and medians lower than one), indicating the absence of, or minimal impairments in these areas; this finding was very similar to that of Balestrieri et al. in their validation study of the Italian version of the Mini-ICF-APP [13]. In our sample, Mini-ICF-APP mean total score was approx. 20, somewhat lower than that observed in the subsample of schizophrenic subjects (mean score approx. 30) in the above cited study [13], and higher than that observed by Molodynski et al. [12] (mean score approx. 14) in a subsample of schizophrenic patients included in the validation study for the English version of the instrument. Bearing in mind that our sample was constituted by a cohort of clinically chronic outpatients who were all on antipsychotic treatment, the somewhat low level of disability reflected by the mean total score at Mini-ICF-APP is not surprising, and is consistent with the English study, which similarly comprised schizophrenic outpatients in attending two community mental health centres, featuring a low-moderate level of severity of symptomatology and functioning, as reflected respectively by mean scores at BPRS (approx. 38) and PSP (approx. 53). On the contrary, the study conducted by Balestrieri et al. [13] included a subsample of schizophrenic patients in the care of a CMHC who were characterized by a more severe clinical status (median CGIs = 6; BPRS median score 75), and of which 40 % were not taking any form of psychopharmacological treatment; this finding may explain, at least in part, the higher mean total score at Mini-ICF-APP observed in this study. With regard to concurrent validity, a high negative correlation was found between mean total score at Mini-ICF-APP (where lower scores indicate better functioning) and mean total score at PSP (where lower scores indicate worse functioning); moreover, a significant negative correlation was found between all Mini-ICF-APP items, with the exception of “mobility”, and mean total score at PSP. When taking into account correlations between each item of Mini-ICF-APP and PSP domains, we found that four items of the former (“adherence to regulations”, “structuring of tasks”, “flexibility”, and “spontaneous activity”) were significantly correlated with three PSP domains (“socially useful activities”, “personal and social relations” and “self-care”). Furthermore, eight items of Mini-ICF-APP (“flexibility”, “competency”, “endurance”, “assertiveness”, “contact with others”, “group integration”, “intimate relationships” “spontaneous activities” and “judgement”) significantly correlated with two PSP domains (“socially useful activities” and “personal and social relations”). The only item of Mini-ICF-APP that failed to show a significant correlation with any PSP domain was “Mobility”, while the PSP domain that failed to correlate with any Mini-ICF-APP item was “aggressive and disturbing behaviour”. Taken together, these findings demonstrate a fairly good concurrent validity with PSP, as previously shown in the validation study for both the Italian [13] and the English versions [12] of the instrument. It should be pointed out that similar results were also obtained by Schaub et al. [38], who found a highly significant correlation between Mini-ICF-APP total score and PSP total score, in a study performed to validate the German version of the instrument in chronic schizophrenic patients.

Although schizophrenic symptomatology and functioning are only partially inter-related, being seen as semi-independent outcome measures [39], and despite the fact that clinical remission does not always imply functional remission [33, 35], significant positive correlations between symptom severity and impaired functioning are generally reported in the literature [1]. In our study, Mini-ICF-APP total scores were significantly correlated with overall scores of both CGI-SCHs and PANSS, a finding indicating a clear relationship between severity of symptomatology and degree of severity of functional deficits measured by Mini-ICF-APP, while no correlations were observed with ratings of positive symptoms evaluated by both CGI-SCHs and PANSS. Lastly, depressive symptomatology and cognitive impairment evaluated by CGI-SCHs, and general psychopathology evaluated by means of PANSS, were all significantly and positively correlated with Mini-ICF-APP total scores. These results are largely in agreement with those of Balestrieri et al. [13]^,^ who found a highly significant correlation between Mini-ICF-APP total score and both CGI-severity score and BPRS global score in their subsample of schizophrenics. Moreover, using BPRS, the same authors found a significant correlation between Mini-ICF-APP total score and several “positive” symptoms (“grandiosity”, “hostility”, “suspiciousness”, “hallucinations”, “unusual thought content”), “mood” symptoms (“guilt”, “elated mood”, “excitement”), and “negative symptoms” (“emotional withdrawal”, “uncooperativeness”). The partial incongruence between the results obtained in our study and those of the study mentioned above [13]^,^ particularly the association between positive symptoms and functioning, may at least in part be explained by the previously cited difference in severity of clinical status between patient samples investigated in the two studies, as the study conducted by our group comprised a fairly stable sample of patients with less severe chronic illness. It should be taken into account that contrasting data are present in literature relating to the role of positive symptoms in disability, while a positive correlation of functioning with negative and depressive symptoms is generally reported [5, 38]. In addition to the correlation observed between severity of cognitive deficits clinically measured using CGI-SCHs cognitive scale and severity of functioning measured by Mini-ICF-APP, we also detected significant correlations with the MMSE and with three out of six BACS domains; this finding was not unexpected, particularly in view of the well established relationship between cognitive and real-world functioning deficits in schizophrenic patients [40]. To establish construct validity, differences in Mini-ICF-APP scores were evaluated, resulting in the expected outcome of significant differences between remitted and recovered patients. Indeed, both clinically and functionally remitted patients displayed a significantly higher Mini-ICF-APP total score compared to unremitted subjects, thus indicating consistently improved functioning; a similar finding was revealed for “recovered” patients, i.e. patients characterized by both clinical and functional remission. Moreover, on analysis, differences in mean scores obtained at each of the thirteen Mini-ICF-APP items provided confirmation of the above findings for a consistent number of items, highlighting better results among remitted and recovered patients for the majority of functioning aspects covered by Mini-ICF-APP. Finally, as expected we found no consistent correlations between Mini-ICF-APP and SWN-S, a result in favour of a fairly good discriminant validity of the instrument.

Before drawing conclusions, several limitations of the present study should be taken into account. In particular, the rather limited size of the study sample, and the prevalent focus on chronic outpatients limit the validity of our findings to the sole patients studied. Additionally, sample heterogeneity should be taken into account, due to inclusion in the study of patients affected by both schizophrenia and schizoaffective disorders. However, it should be underlined that in our cohort no differences in sociodemographic and clinical parameters were detected between the two diagnostic subsamples. However, even in the light of these limitations, the evidence obtained would seem to be of interest for clinicians.

## Conclusions

Results obtained in this study show that the Mini-ICF-APP is a valid measure of functioning for use in chronic patients affected by schizophrenia and schizoaffective disorders. Moreover, the tool holds characteristics which are not available in other instruments [13], which specifically enable the measurement of “capacities”, and addresses aspects of functioning of particular relevance to the working environment [12]. Therefore, in our opinion, Mini-ICF-APP should be given due recognition and added to the scenario of existing instruments intended for use in evaluating functioning in the mentally ill. Moreover, the user-friendly and streamlined application of the instrument is particularly suited for use in a routine psychiatric setting.
